# Three-Dimensional Modeling of Maize Canopies Based on Computational Intelligence

**DOI:** 10.34133/plantphenomics.0160

**Published:** 2024-03-20

**Authors:** Yandong Wu, Weiliang Wen, Shenghao Gu, Guanmin Huang, Chuanyu Wang, Xianju Lu, Pengliang Xiao, Xinyu Guo, Linsheng Huang

**Affiliations:** ^1^ National Engineering Research Center for Agro-Ecological Big Data Analysis & Application, Anhui University, Hefei 230601, China.; ^2^ Information Technology Research Center, Beijing Academy of Agriculture and Forestry Sciences, Beijing 100097, China.; ^3^ Beijing Key Lab of Digital Plant, National Engineering Research Center for Information Technology in Agriculture, Beijing 100097, China.; ^4^ Nongxin Science & Technology (Beijing) Co., Ltd, Beijing 100097, China.

## Abstract

The 3-dimensional (3D) modeling of crop canopies is fundamental for studying functional-structural plant models. Existing studies often fail to capture the structural characteristics of crop canopies, such as organ overlapping and resource competition. To address this issue, we propose a 3D maize modeling method based on computational intelligence. An initial 3D maize canopy is created using the t-distribution method to reflect characteristics of the plant architecture. The subsequent model considers the 3D phytomers of maize as intelligent agents. The aim is to maximize the ratio of sunlit leaf area, and by iteratively modifying the azimuth angle of the 3D phytomers, a 3D maize canopy model that maximizes light resource interception can be constructed. Additionally, the method incorporates a reflective approach to optimize the canopy and utilizes a mesh deformation technique for detecting and responding to leaf collisions within the canopy. Six canopy models of 2 varieties plus 3 planting densities was constructed for validation. The average *R*^2^ of the difference in azimuth angle between adjacent leaves is 0.71, with a canopy coverage error range of 7% to 17%. Another 3D maize canopy model constructed using 12 distinct density gradients demonstrates the proportion of leaves perpendicular to the row direction increases along with the density. The proportion of these leaves steadily increased after 9 × 10^4^ plants ha^−1^. This study presents a 3D modeling method for the maize canopy. It is a beneficial exploration of swarm intelligence on crops and generates a new way for exploring efficient resources utilization of crop canopies.

## Introduction

The structure of crop canopies plays a crucial role in fulfilling the functional requirements of crop production. The morphological and structural features of crop canopies have always been fundamental in the way crop scientists understand, analyze, and evaluate crops. The structure of crop canopies meaningfully impacts the crop's resource utilization efficiency, yield, and stress resistance. The study of crop canopy structure has become an integral part of crop cultivation, crop phenomics [1,2], and functional-structural plant models (FSPMs) [3,4], holding substantial implications for crop ideotype breeding [5], high-density planting for increased yield [6,7], and improved light efficiency [8].

However, since crop canopy structure exhibits a complex spatial distribution, demonstrates pronounced spatial variability in organ morphology, and encompasses considerable internal overlapping and interaction, the canopy formation process extends beyond a mere physical replication of individual plants [9]. Owing to these constraints and lack of algorithms, technology, and a comprehensive technical system, the construction of a 3-dimensional (3D) crop canopy model that captures the diversity, density, and cultivation management practices has posed considerable challenges in this field [10].

With regard to phenotypic data acquisition and analysis of crop canopy structure, sensors mounted on drones, such as visible light or LiDAR, can be used to gather morphological structure data of crop canopies, allowing for the assessment of phenotypic indicators like plant height [11], coverage [12], and biomass. The top-view images of crop canopies, through segmentation of early-stage images, facilitate the positioning of plants within the population [13]. However, due to the considerable distance of drone platforms from the crop canopies, their instability, and low imaging resolution, they primarily capture the external contour data of the crop canopy, making it difficult to acquire high-resolution internal morphological structure information. Field-based phenotyping platforms [14] enhance the stability and resolution of crop canopy data collection and also provide time-series data of crop canopy growth, thereby improving the accuracy of crop canopy structure phenotype analysis. For example, Li et al. [15] used a field-based phenotyping platform to obtain time-series data of crop canopies, and through the integration of image and point cloud data, they were able to segment plants and organs within the population and extract phenotypic parameters such as plant height, leaf inclination, and azimuth angles. However, due to the high construction and maintenance costs, limited range of action, and difficulty in practical field production application of the phenotyping platforms, their use in analyzing the phenotypic structure of crop canopies remains limited. Xiao et al. [16] achieved low-cost acquisition and analysis of 3D point clouds of crop canopies through multiangle imaging with low-altitude drones, but this stereo vision–based method still fails to address the problem of internal overlapping in the population. Solving the problem of internal occlusion in plant populations, particularly for densely planted plants, is challenging when relying solely on measured data. It is essential to combine data with knowledge and solve it through 3D modeling.

In terms of constructing 3D maize canopy model structure, electromagnetic digitizers are used to manually obtain 3D coordinate points of each plant and organ within the maize canopy, enabling in situ 3D reconstruction. While this method is highly accurate and convenient for subsequent applications due to the inclusion of semantic information, its efficiency is extremely low, making it challenging to realize large-scale canopy 3D reconstruction [17]. Multiangle imaging methods, which involve acquiring multiangle images of maize canopies using drones or manual photography and constructing 3D point clouds based on multiangle 3D reconstruction, are mainly suitable for 3D reconstruction of maize canopies before ridging or in plots with marked spatial separation [16,18]. FSPM-based methods [19] integrate maize growth and development models with measured data to construct 3D models of maize canopies, simulating the 3D structure and photosynthetic production material distribution of maize canopies. However, these models struggle to reflect differences in plant types and canopy structure morphology. Statistical plant type–based 3D modeling methods for maize canopies involve measuring plant type parameters of a few target plants within the population, constructing a statistical model of plant type parameters, and combining parametric modeling methods to achieve 3D modeling of maize canopies [9]. Such methods can statistically reflect morphological differences due to variety plant types and cultivation management measures but still fail to capture population characteristics like internal overlapping and resource competition. Interactive modeling methods, despite allowing for adjustments in prebuilt population parameters, require extensive manual interaction and are inefficient. Crop population 3D models constructed using software like GroIMP struggle to reflect variety characteristics and primarily rely on individual plant replication. Overall, the construction of 3D models of crop populations in practical applications still primarily revolves around plant replication [20], and the constructed models struggle to reflect characteristics such as resource competition and interaction caused by overlapping within the population. Details such as the orientation of plants within the population and the growth position and orientation of leaves are insufficiently considered, and the randomness in simulation noticeably impacts subsequent calculations such as canopy light distribution, hindering the improvement of accuracy in FSPMs.

With the rapid development of computer hardware and software, computational intelligence (CI) has advanced rapidly in recent years, leveraging large-scale computation to mine domain knowledge and develop various new methods for solving complex problems. CI primarily includes 5 methods: fuzzy logic, probabilistic approaches, swarm intelligence, neural networks, and evolutionary computation [21]. In agriculture, CI has been widely applied in predicting and detecting crop diseases, analyzing soil and climate data, and optimizing crop yields. For instance, intelligent decision systems and light detection technologies allow researchers to predict disease rates and thereby improve crop yield productivity [22]. The formation of maize canopy structure is a complex process, where organs within the population influence each other to compete for more resources, exhibiting characteristics of swarm intelligence. However, to date, there has been no report of utilizing CI to solve the construction of 3D models of maize canopies. The leaves in the plant population can be viewed as agents that independently adjust their position based on resource competition and the positional relationship between adjacent organs. With this in mind, we aim to incorporate CI into 3D modeling of plant canopies.

This paper takes maize canopies as an example and starts with the 3D phytomer [23] of maize, using the intelligent computation of the azimuth angles of 3D phytomers within each plant in the maize canopy. Through focusing on maximizing light interception during key growth stages as the optimization goal, we aim to construct an algorithm for 3D modeling of maize canopies that can reflect the differences in varieties and cultivation management measures.

## Materials and Methods

### Experimental design and data acquisition

#### Experimental design

The experiment was conducted in 2022 at the field-based phenotyping platform of the Beijing Academy of Agriculture and Forestry Sciences (39°56' N, 116°16' E). Two maize varieties, Jingnongke728 (JNK728) and Jingke968 (JK968), were selected for the study. The plants were sown on 2022 June 15, with planting densities set at 3, 6, and 9 × 10^4^ plants ha^−1^, creating a total of 6 plots. The row spacing was maintained at 60 cm, oriented in an east-west direction. Throughout the whole growth period, drip irrigation was employed to ensure adequate water and nutrient supply [24].

#### Data acquisition

Data collection was carried out during the stable phase of maize plant structure and canopy formation, specifically during the R3 stage (milk stage). This involved utilizing the field-based phenotyping platform [14,15] to capture top-view images and 3D point clouds of each plot, aiding in the verification of maize canopy models. Within each plot, a 3 ×3 grid of 9 plants was selected for measurement. The azimuth angle of all leaves on each plant was recorded to evaluate the simulation results of the model. These 9 plants were then relocated indoors for further analysis. Using the multiview phenotyping platform MVS-Pheno [25], multiangle image data were gathered, and plant point clouds were generated to extract phenotypic parameters of the plants. These parameters served as initial plant architecture inputs for the construction of maize canopy models. Finally, a Fastrak (Polhemus, Colchester, VT, USA) 3D digitizer was used to collect 3D phytomer data of the plants, contributing to the development of a 3D phytomer database for each variety, which was used as a template for the 3D modeling of maize canopies [23,26].

### 3D modeling of maize canopies based on CI

#### Overview of the method

**Fig. 1.**
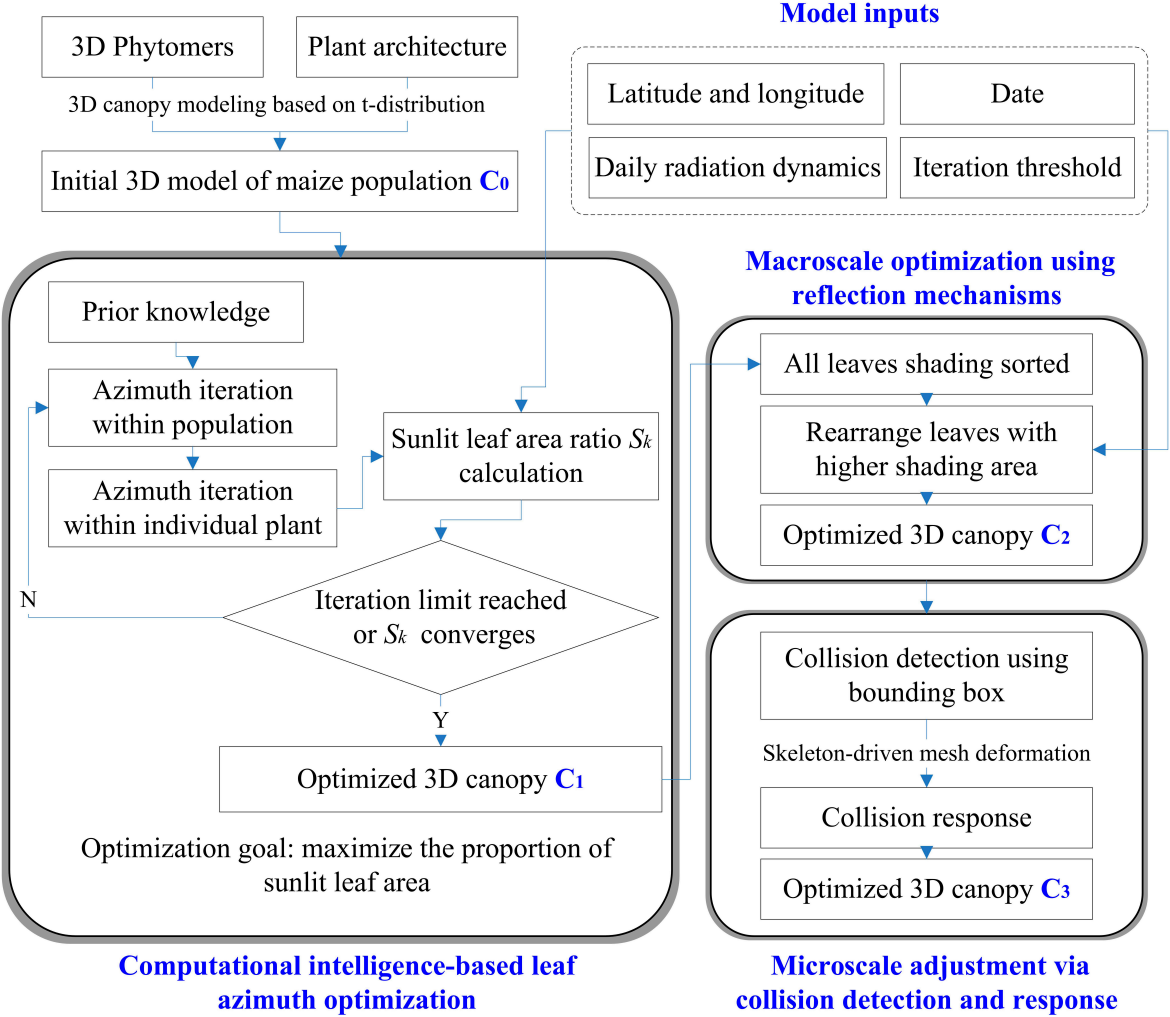
The complete methodological process. C_0_: The baseline canopy model for the subsequent iterative computations. C_1_: The initially optimized maize canopy model based on C_0_. C_2_: Optimized canopy model based on C_1_ using reflective approach-based optimization. C_3_: Optimized canopy model based on C_2_ by employing detection and response to organ collisions method.

This methodology comprises 5 key steps (Fig. 1): generation of original 3D maize canopy model, computation of the sunlit leaf area index, optimization of leaf azimuth angles using CI, reflective optimization of canopy structure, and detection and response to organ collisions.

1. Generation of original 3D maize canopy model. This initial phase involves creating a 3D maize canopy model, which is based on the 3D phytomer of maize, incorporating a maize canopy 3D modeling approach grounded in the t-distribution function, and taking into account the row spacing and plant numbers in the target maize canopy.

2. Computation of sunlit leaf area index. This step introduces a method to calculate the sunlit leaf area index, focusing on maximizing direct light resource utilization as the optimization target for further calculations.

3. Optimization of leaf azimuth angle using CI. With the objective of maximizing the leaf area, this step involves iterative C_0_ calculations and optimization of the azimuth angles of each 3D phytomer within the original maize canopy, culminating in the formulation of an initially optimized maize canopy model C_1_.

4. Reflective optimization of canopy structure. Following the initial optimization, a reflection-based optimization process is conducted, focusing on the entirety of the maize canopy, ultimately resulting in the development of a reflected model C_2_.

5. Detection and response to organ collisions. The final step encompasses the refinement of the reflected 3D maize canopy model C_2_, primarily addressing internal organ collisions within the canopy and subtly adjusting these structures to enhance model realism, thereby leading to the formulation of the ultimate 3D maize canopy model C_3_.

#### Generation of original 3D maize canopy model

Building on prior 3D modeling of the maize canopy efforts, this study uses established 3D maize phytomers [23] and a 3D modeling of the maize canopy approach based on t-distribution [9] to develop the original 3D model C_0_. A 3D maize phytomer here refers to a meticulously crafted 3D mesh model including components such as leaves, leaf sheaths, nodes, internodes, and associated appendages like tassels. These phytomers represent the fundamental modular units of maize morphology, exhibiting 3D spatial variability influenced by varietal characteristics, phyllotaxy (leaf arrangement), and agronomic practices. The t-distribution-based method integrates morphometric data from maize (such as height, leaf count, leaf length, leaf inclination, and azimuth angle) obtained via the MVS-Pheno platform. By using similarity functions, it selects corresponding 3D phytomer templates, constructing a statistically representative 3D maize canopy model.

#### Sunlit leaf area ratio calculation

The capacity to outcompete for light is a critical adaptive strategy in crop canopy self-organization and regulation. Emphasizing this, maximizing light interception efficiency is posited as the primary objective in optimizing the 3D maize canopy model C_0_. Considering the dominance of direct sunlight within the canopy and its considerable variability across different heights, leaves, and leaf positions (in contrast to the relatively uniform distribution of diffuse light), the study proposes the sunlit leaf area ratio as a pivotal optimization metric.

**Fig. 2.**
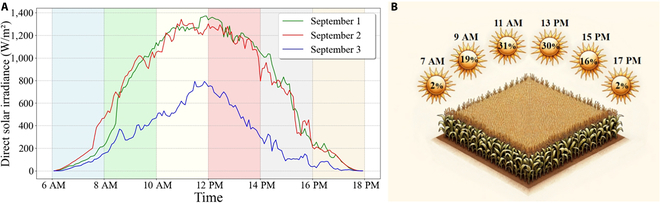
Radiation dynamics and weight proportions in Beijing over a 3-d period. A 3-d average of radiation dynamics in Beijing from 2022 September 1 to 3, used for calculating weights (A), a representation of radiation weight proportions across different time intervals (B).

Given the variability in solar incidence angles across different maize-growing regions and throughout the day, the area of leaves illuminated by direct sunlight within the canopy fluctuates [8,27]. The optimization goal is thus to maximize the direct light interception leaf area under clear sky conditions during the grain-filling stage. Using Beijing as a case study, based on solar radiation data during clear sky conditions in the grain-filling stage (Fig. 2A), and considering the considerable variation in radiation intensity throughout the day, the daylight hours are segmented into discrete intervals for computational efficiency. For instance, a day is divided into 6 intervals (Fig. 2B), with each interval assigned a radiation weighting based on its specific solar radiation profile. The radiation intensity at time *t* is represented as *I_t_*, and the total direct sunlight intensity for the day is *I_all_*. The radiation weighting for a given moment is calculated as follows:

$$\omega_{t}=\frac{\int_{T_{t}}^{T_{t+2}}I_{t}\mathrm{dt}}{I_{\mathrm{all}}}$$ (1)

Subsequently, the illuminated leaf area within the canopy at each time interval is calculated and aggregated using weighted summation, facilitating the computation of the sunlit leaf area ratio *S_k_* in the *k* iteration of 3D modeling of the maize canopy.

To calculate the area of unobstructed triangular facets within the 3D phytomers of the maize canopy, these facets need to be projected from 3D space onto a 2-dimensional plane at varying angles of solar incidence. Initially, a large grid area, perpendicular to the solar incidence plane, is established and then uniformly divided into *m* smaller grids. Within each of these smaller grids, the triangular facets are sorted based on the proximity of their centroid's Z-axis coordinate to the plane. The triangular facet closest to the plane in each grid is selected, representing the area of the unobstructed triangular facet within grid *i* at time *t,S*_*area*[*i*, *t*]_.

The total leaf area of the canopy is denoted as *S_all_*. After the initial establishment of the canopy model, subsequent iterations primarily involve adjusting the azimuth angles of the various 3D phytomers within the canopy; hence, the total leaf area *S_all_* remains constant. *ω_t_* represents the radiation intensity weight at the current moment, and *R* represent the collection of all time points throughout the day. The method for calculating the proportion of sunlit leaf area is then defined as follows:

$$S_{k}=\sum_{t\in R}\omega_{t}\times \frac{\sum_{i=1}^{m}S_{\text{area}\left[i,t\right]}}{S_{\mathrm{all}}}$$ (2)

#### Optimization of leaf azimuth angle based on CI

**Fig. 3.**
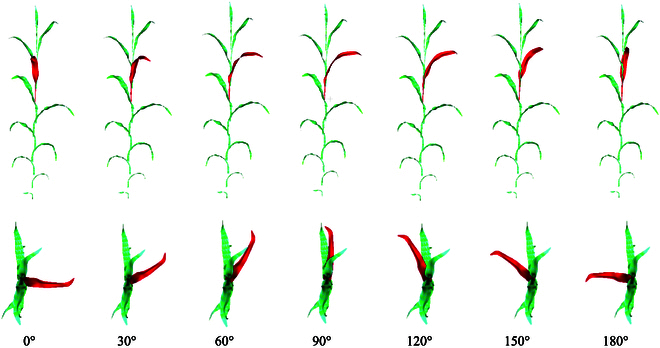
A schematic of the rotation process, with the red portion indicating the rotating phytomers.

The essence of the azimuth angle iteration process is the continuous counterclockwise rotation of the 3D phytomer mesh model around the stem (Z-axis), generating various 3D maize canopy models. The process of rotation is illustrated in Fig. 3.

Maize canopy contains multiple plants, each comprising several 3D phytomers. Therefore, the sequence of adjusting the 3D phytomers is crucial. The plant adjustment strategy must ensure that all plants are modified while minimizing the impact of each plant being adjusted on the already modified plants. To achieve this, the following strategy is established to determine the order of adjustment for plants and the 3D phytomers within them: starting with the first/last plants on the boundary row within the planting area, adjust all the plants along the boundary line in a clockwise direction, gradually moving inwards until all plants are adjusted (Fig. S1A). At the level of 3D phytomers within plants, due to the vertical growth structure of maize, the higher phytomers have a greater shading effect on the lower ones. Therefore, the azimuth angle of the highest phytomer is set as the reference for adjustments, progressing from top to bottom (Fig. S1B), ensuring each layer of phytomers receives maximal direct sunlight. Existing prior knowledge indicates that the difference between adjacent azimuth angles typically ranges between 90° and 270°. This provides a reference range for azimuth adjustments, enhancing realism while reducing computational load during optimization. Each iteration's azimuth adjustment is based on the results of the previous adjustment.

Beyond the order of iterations, the step size of the azimuth angle iteration directly affects the method's precision and computational load. If the step size is too small, the change of *S_k_* may be negligible, marked increasing the computational load. Conversely, if the step size is too large, the change of *S_k_* may be too drastic, hindering the convergence of the iteration process. Therefore, after extensive experimental testing and analysis, the azimuth angle iteration step size is set to 5°. At this step size, each iteration effectively alters the value size without causing excessive fluctuations in the projected area, thus preventing large oscillations.

The pseudo-code for the entire iterative process is shown in the algorithm in Fig. S2. There are 2 ways to terminate the iteration during the process. One is by comparing the change of *S_k_* with a predefined threshold *Q_m_*. If the change of *S_k_* is consistently below this threshold over multiple iterations, the model is deemed to have converged, and the iteration is terminated. The other method sets an upper limit on the number of iterations to prevent the algorithm from running indefinitely in search of a potentially nonexistent "perfect" solution, ensuring that the algorithm completes within a reasonable time.

After the aforementioned iterative and optimization process, the azimuth angle data of the 3D phytomers at the convergence of *S_k_* is taken as the final result. This azimuth angle data reflects the optimized state of the 3D maize canopy model for maximal direct sunlight interception. The resulting model C_1_ represents an initially optimized 3D maize canopy model that more accurately reflects the overall characteristics of the maize canopy.

#### Reflective approach-based optimization

The previously described iterative optimization of leaf azimuth angles adjusts individual plants and 3D phytomers within the canopy. Although this takes into account the factors of shading and avoidance in local areas, it lacks a consideration of the overall characteristics of the canopy. To address this, a reflective approach, inspired by generative artificial intelligence concepts [28], is introduced to further optimize the overall 3D maize canopy model. This approach is a self-adjusting process that reflects and adjusts the model's accuracy and effectiveness after each simulation. Considering upcoming organ collision detection and response, and referencing the dual-scale automaton approach [29], this reflective approach can be seen as an optimization of a macro state, while the subsequent organ collision detection and response is viewed as a micro-state adjustment.

From a holistic perspective of maize canopies, adjustments are no longer made simply in order from the top to the bottom of the canopy. Instead, a more comprehensive approach is taken, where all 3D phytomers within the canopy are sorted based on their shading, and quantitative adjustments are made. This method allows for higher-level optimization of the 3D maize canopy model, better reflecting the overall characteristics of the canopy.

Specifically, the process begins by setting the angle of incident light as vertically downward and using the initially optimized 3D maize canopy model C_1_ as input. The optimization follows these steps:

1. Calculate the area ratio of the shaded part for each 3D phytomer.

2. Sort all 3D phytomers based on the proportion of the shaded area. This identifies which phytomers are most and least likely to be shaded.

3. Select the top 50% of phytomers with the largest shaded area for azimuth angle adjustment, ensuring priority adjustment for those most prone to shading. This step also takes into account previous overall azimuth angle adjustments to maintain consistency and avoid conflicts or redundancy. Each phytomer will have a maximum adjustment threshold of 30°, with iterative steps of 2°.

4. During the iteration process, select the azimuth angle data that maximizes the sunlit leaf area ratio of the 3D maize canopy model for the next round of iteration.

5. The optimization of the canopy model is considered complete when a preset iteration limit is reached or the sunlit leaf area ratio of the 3D maize canopy model stabilizes. If these conditions are not met, the process returns to the first step, using new azimuth angle data for optimization. This reflective approach of fine-tuning from a macro perspective ensures a more objective and comprehensive optimization of the 3D maize canopy model C_2_, thereby better reflecting the overall characteristics of the canopy.

#### Organ collision detection and response

In actual maize canopies, spatial adjustments occur due to leaf collisions, a situation exacerbated by increasing density. This issue is also considered in the generated 3D maize canopy model. To address this, collision detection and mesh deformation methods are introduced. Compared with the overall canopy perspective C_2_ described earlier, this step primarily addresses the detailed issues at the organ scale, specifically within individual 3D phytomers, and is therefore considered a micro-state adjustment C_3_.

Preliminary collision detection is performed using bounding boxes [30]. To expedite the process, the axis-aligned bounding boxes of each maize 3D phytomer are first calculated. If the axis-aligned bounding boxes of two 3D phytomers do not intersect, then these phytomers do not overlap and cannot collide. If they intersect, the Separating Axis Theorem [31,32] is further employed for collision detection, which involves checking whether the projections of the two 3D phytomers overlap on all possible axes. A collision is confirmed only if the projections overlap on all axes.

Upon detecting a collision between two 3D phytomers, a mesh deformation method [33] is employed for collision response. In actual canopies, leaves farther from the stem are more likely to be displaced by external forces upon collision. Assuming that a leaf on a 3D phytomer comprises *M* vein points, the first *m* points near the stem do not deform within the unit. The remaining *M−m* points are used as the driven point set for deformation, guided by the vein-driven mesh deformation method for leaf deformation. The weights and positions of the vein-driven points can be updated using the following Eqs. 3 and 4:

wi0,0≤i≤mi−m/nall,m<i≤M (3)

$$P_{i}^{\mathrm{new}}=P_{i}^{\mathrm{ori}}+w_{i}\times d_{\mathrm{off}}$$ (4)

**Fig. 4.**
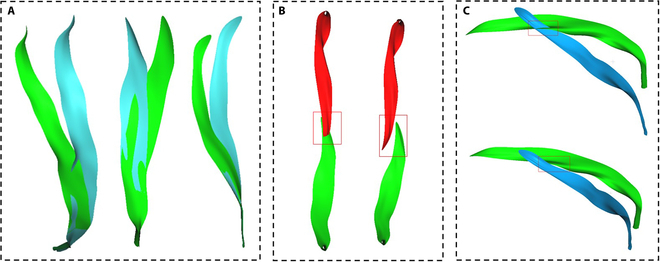
Schematic of organ collision response. The mesh deformation of the leaf (A), with the blue part representing the leaf before deformation and the green part after, the results before and after collision deformation of maize leaves from different plants (B), the outcomes before and after collision deformation optimization of leaves at different growth heights within the same plant (C), with the upper image showing the state before optimization and the lower image after.

In this formula, *w_i_* represents the weight value, *i* is the index of the current leaf vein point, *n_all_* is the total number of points on the leaf of the 3D phytomer, $P_{i}^{\mathrm{ori}}$and $P_{i}^{\mathrm{new}}$ are the coordinates of the *i-th* point before and after the update, $P_{i}^{\mathrm{ori}}$ is the original position, and *d_off_* is the offset. The deformation of the leaf in response to a collision is illustrated in Fig. 4A, while Fig. 4B and C show comparative images before and after collision detection and response in 2 different scenarios.

#### Evaluating indicators

As this method simulates field maize canopies rather than reconstructing them, verifying its effectiveness is crucial. To evaluate the method, the following 3 indicators are proposed, focusing on the leaf azimuth angle, a key aspect of this study.

As it is difficult to ensure that the orientation of plants in the constructed canopy exactly corresponds to actual plants in a real community, the difference in azimuth angles of adjacent leaves on a plant is used as a relative value to evaluate the azimuth angle simulation of the 3D canopy model. If the azimuth angle of the *i-th* leaf on a plant is denoted as *φ_i_*, then the formula for calculating the difference in azimuth angles of adjacent leaves on a plant ∆*φ_i_* is as follows:

$$∆\varphi_{i}=\left|\varphi_{i+1}-\varphi_{i}\right|\;$$ (5)

With increased planting density in maize, competition for light resources intensifies, and one plasticity approach is the directional adjustment of leaf azimuth. Therefore, to qualitatively validate the accuracy of the 3D modeling of the maize canopy, the proportion of leaves oriented perpendicular to the row direction in maize plants at different densities is evaluated [33,34]. If the total number of phytomers is *N_all_*, and the number of phytomers with azimuth angles (assuming the row direction is north-south) between 60° and 120° is *N_vert_*, then the formula for calculating the proportion *p* of leaf azimuth angles perpendicular to the row direction is as follows:

$$p=\left(N_{\text{vert}}/N_{\mathrm{all}}\right)\times 100\%$$ (6)

Canopy coverage is the percentage of the crop canopy's vertical projection area on the ground relative to the total area of the assessment region. It quantifies the density of vegetation and reflects the growth status of the vegetation [35,36]. Canopy coverage is used as the third evaluation indicator.

## Results

### Visualization of the 3D canopy models

Utilizing the CI-based 3D modeling method for maize canopies, 3D models of 6 plots for the JNK728 and JK968 varieties were constructed at densities of 3, 6, and 9 ×10^4^ plants ha^−1^. The planting strategy of each plot includes 25 plants arranged in a 5 × 5 grid, with a row spacing of 60 cm, as illustrated in Fig. S3.

**Fig. 5.**
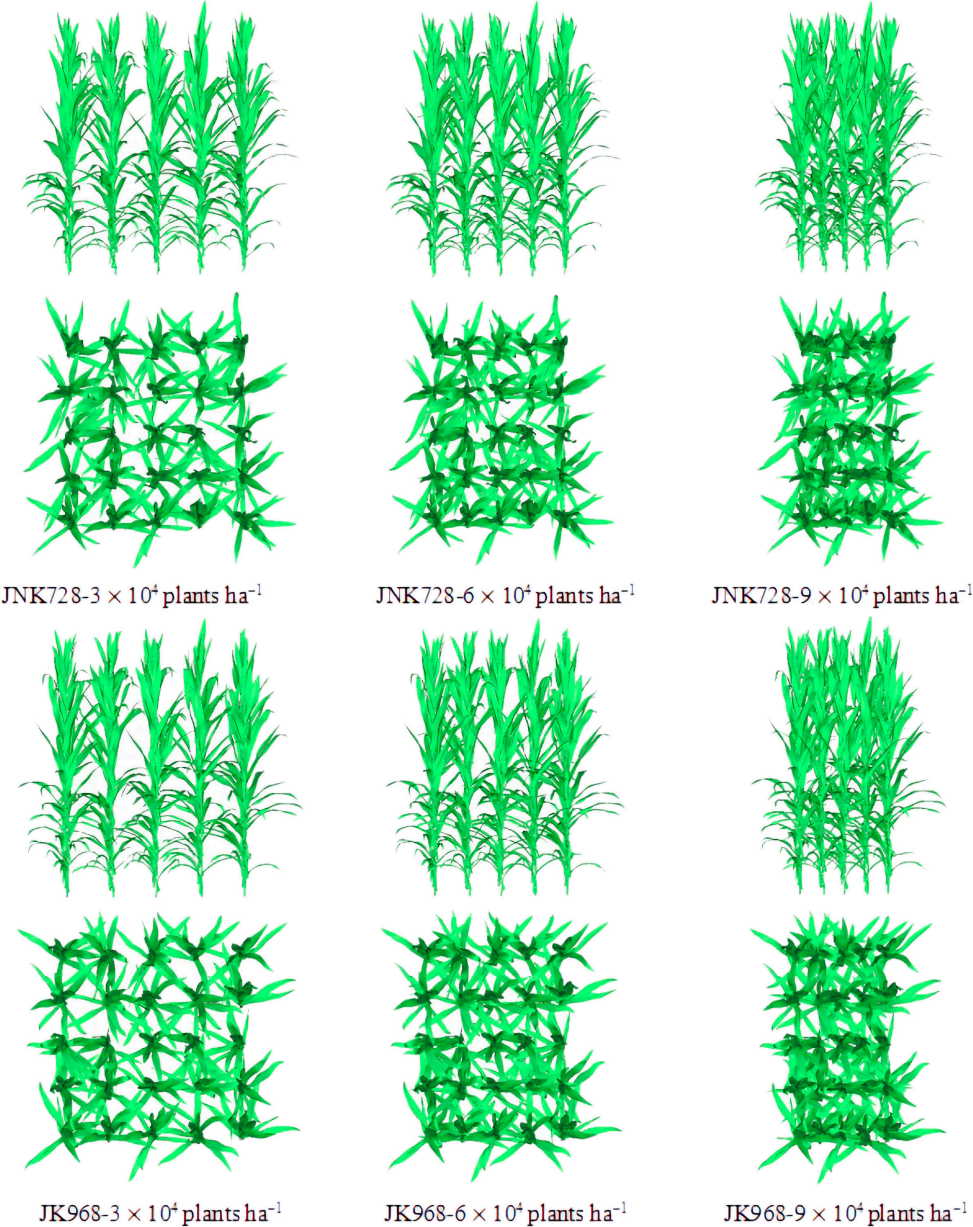
The front and top views of the 3D maize canopy models of C_3_ in different varieties and densities constructed using the proposed method. The angle of view is perpendicular to the row direction.

The visualization effects of the 6 maize canopies C_3_ constructed using the method presented in this paper are shown in Fig. 5. With increasing planting density, the space between plants becomes narrower, leading to more pronounced shading between them. The visualization results indicate that plants on the edges of the canopy, experiencing less shading, have a similar and more dispersed distribution of external leaf azimuth angles across different densities within the same variety. This reflects the method's ability to capture the marginal effects in maize canopies. Additionally, internal leaves within the canopy adjust their azimuth angles to compete for more light resources as the density increases.

### Validation results

#### Validation of differences in leaf azimuth angles between adjacent leaves on individual plants

**Fig. 6.**
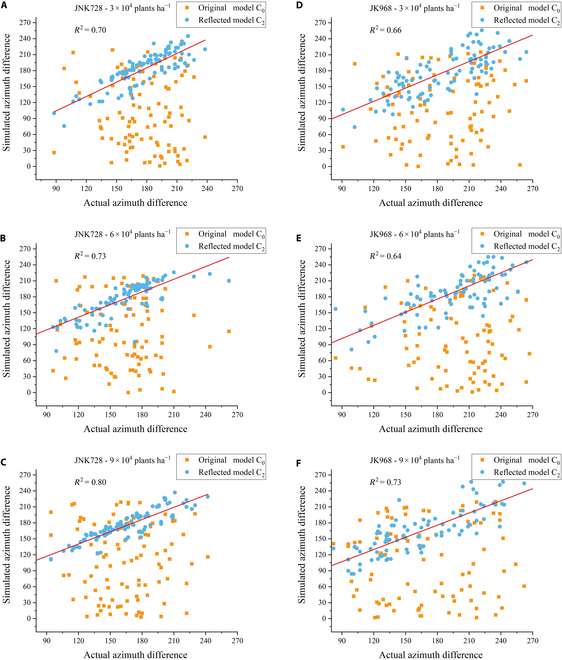
Comparative results of the differences in azimuth angles of adjacent leaves between the 3D models of maize canopy constructed under different varieties and planting densities and the actual maize canopy in the field. The comparison results between the azimuth angle differences of adjacent leaves on maize plants in 3D canopy models (C_0_, C_2_) of different varieties and densities and those in actual field canopies. (A to C) are of JNK728 and (D to F) are of JK968 in different planting densities.

Since the field canopy includes azimuth angle data for 9 plants, and the model generated data for 25 plants, the 9 central plants of the model were selected as a control canopy. The differences in azimuth angles of adjacent leaves on plants in the maize canopy ∆*φ_i_* were compared and analyzed, with results shown in Fig. 6. The results indicate that the difference in azimuth angles of adjacent leaves in original 3D maize canopy model C_0_, compared to the measured data, were not well represented and were more dispersed. In contrast, the optimized 3D maize canopy model C_2_ showed a certain degree of consistency with the measured data. The *R*^2^ for the 6 canopies were 0.70, 0.73, 0.80, 0.66, 0.64, and 0.73, respectively. This suggests that the 3D maize canopy model constructed using this method can more accurately simulate the distribution pattern of the differences in adjacent leaf azimuth angles on plants within field maize canopies.

**Fig. 7.**
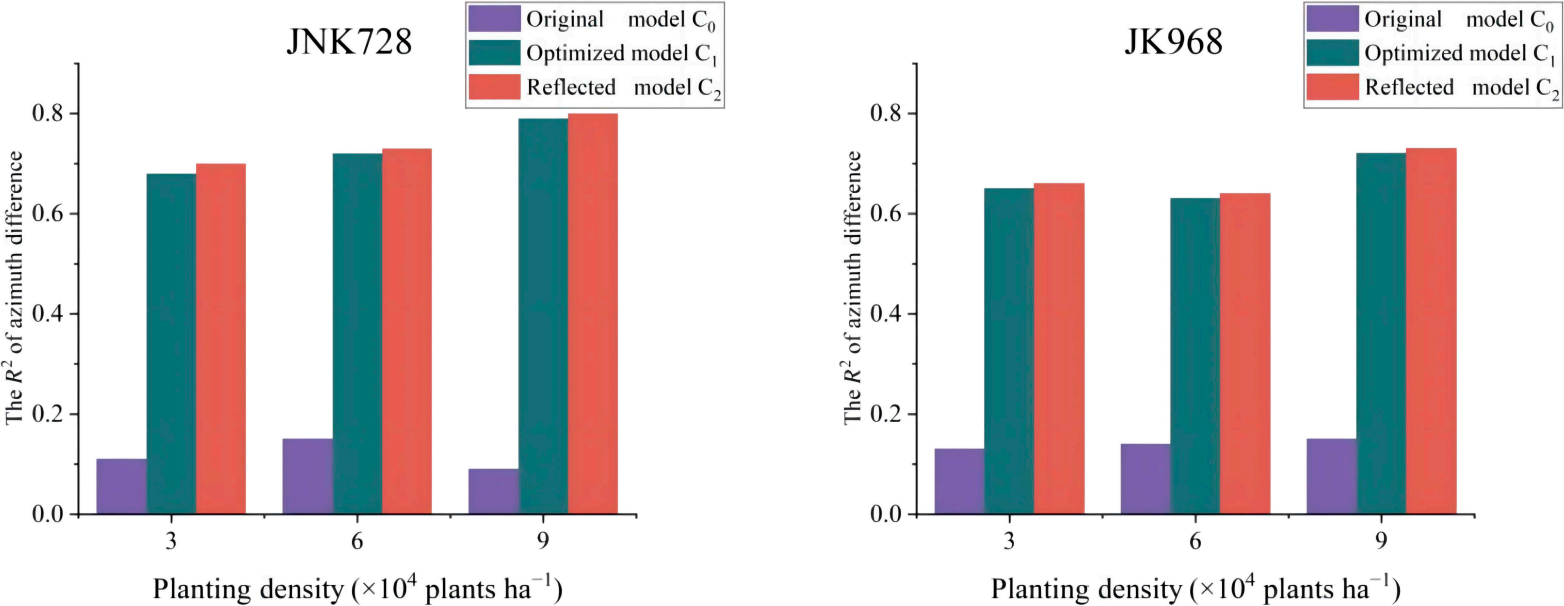
The changes in the differences in adjacent leaf azimuth angles in the original model C_0_, the algorithm-optimized model C_1_, and the reflective approach-enhanced model C_2_.

For the JNK728 and JK968 varieties in the 6 plots, the differences in adjacent leaf azimuth angles in the original 3D canopy model C_0_, constructed using the t-distribution, showed substantial randomness, with a consistency *R*^2^ of around 0.2 compared to the measured data. After optimization of the canopy model C_1_ using the presented method, the *R*^2^ improved from below 0.2 to above 0.6, particularly for the JNK728 variety at 9 × 10^4^ plants h*a*^−1^, reaching as high as 0.79. Moreover, after applying the reflective approach, all plot models C_2_ saw a slight increase in *R*^2^, with the JNK728 variety at 9 × 10^4^ plants ha^−1^ showing an accuracy improvement to above 0.8. This indicates the effectiveness of the reflective approach in enhancing model precision (Fig. 7).

#### Canopy coverage validation

**Fig. 8.**
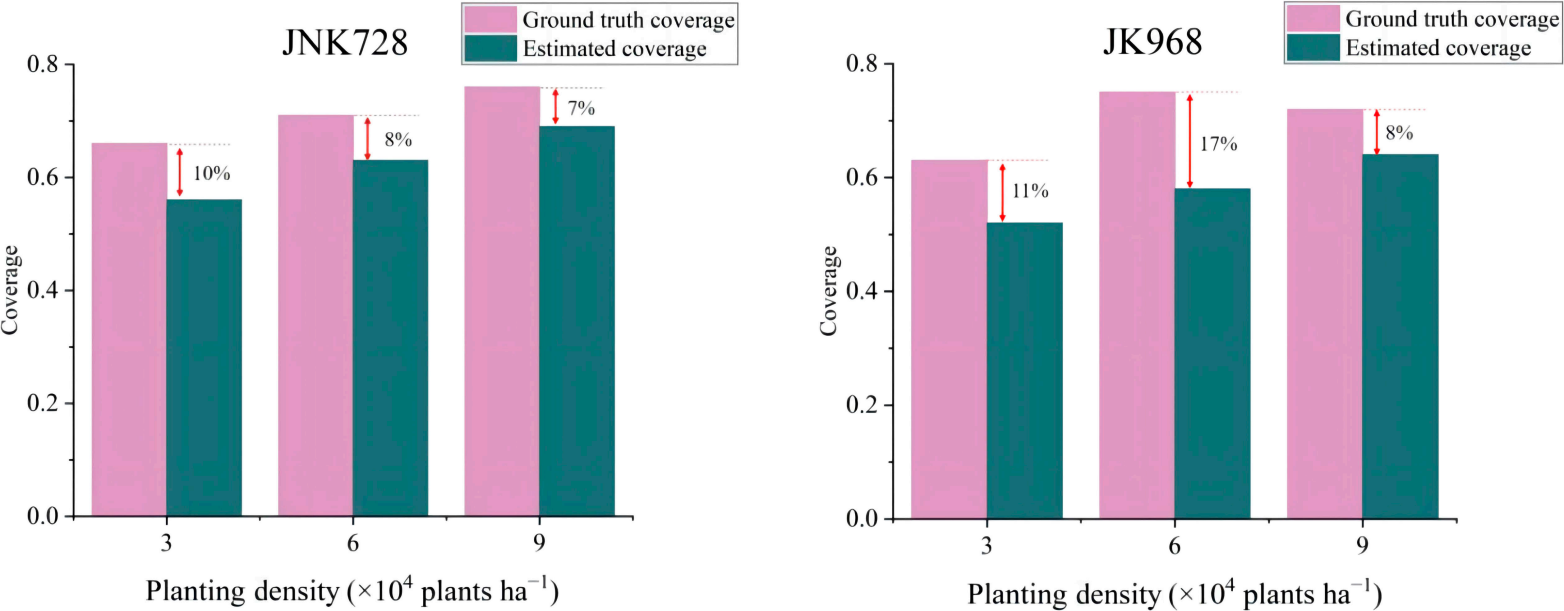
The comparison of the measured and simulated canopy coverage.

Top-view images of each plot were obtained using the rail-based phenotyping platform, and canopy coverage was extracted for evaluating the accuracy of the community characteristics of the constructed 3D models, as shown in Fig. 8. With increasing planting density, the canopy coverage data for both varieties increased, consistent with actual canopy conditions. The JNK728 variety had smaller canopy coverage errors across all planting densities, with the smallest error of 7% at 9 × 10^4^ plants ha^−1^. The errors for the JK968 variety at the same densities were higher than those for JNK728 but did not exceed 17%. The overall lower canopy coverage values in the 3D models compared to those extracted from images could be partly attributed to the presence of weeds and fallen leaves on the actual field, which would increase the measured canopy coverage. As the maize canopy matures, its lower leaves eventually detach from the main stem and fall to the ground. In a 3D model, these fallen leaves are not accurately represented. Top-view imagery may capture fallen leaves and weeds as part of the canopy coverage, inflating the image-based measurement.

#### Proportion of leaf azimuth angles perpendicular to the row direction

**Fig. 9.**
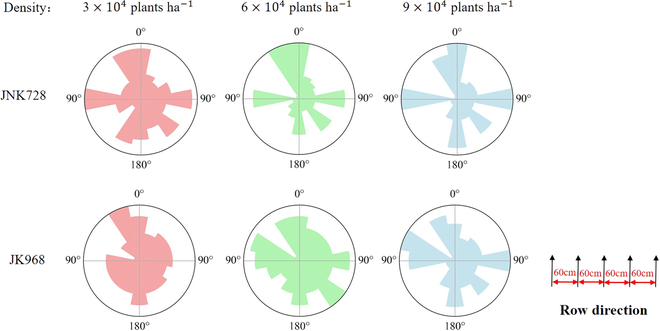
Distribution maps of leaf azimuth angles for JK968 and JNK728 at different planting densities.

The distribution proportions of leaf azimuth angles in the 6 constructed maize canopies are shown in Fig. 9. For the JNK728 variety, at a density of 3 × 10^4^ plants ha^−1^, the distribution of plant azimuth angles within the canopy was relatively balanced, with the proportion *p* near 90° reaching 37.8%. At 6 × 10^4^ plants ha^−1^, this proportion *p* slightly decreased to 35.6%, and at 9 × 10^4^ plants ha^−1^, *p* increased to 40.2%. For the JK968 variety, at 3 × 10^4^ plants ha^−1^, the proportion *p* near 90° was 24.7%; however, this proportion *p* marked increased to 50.5% at 6 × 10^4^ plants ha^−1^ and further rose to 56.9% at 9 ×10^4^ plants ha^−1^.

From these observations, despite the more dispersed leaf azimuth angles in the JNK728 and JK968 varieties at lower densities, both varieties exhibited an increasing trend in the proportion of leaf orientation toward 90° as planting density increased. This phenomenon suggests that, in response to the environmental stress of high-density planting, leaves of both varieties tend to grow more perpendicular to the row direction.

### Maize canopy modeling for gradient planting densities

Since our method can simulate the variation of leaf azimuth angles within maize canopies under different planting densities, we referenced the density stress experiment conducted by Wang et al. [37] in Qitai, Xinjiang, China (43°29′N, 89°28′E), and used the 3D leaf template of maize variety [17] Xianyu335 to construct 3D maize canopy models across a density gradient. The canopies were planted following maize densities ranging from 1.5 to 18 × 10^4^ plants ha^−1^, with an incremental step of 1.5 × 10^4^ plants ha^−1^, resulting in a total of 12 different density plots. The planting patterns were based on alternate row spacings of 70 and 40 cm, representing wide and narrow rows, respectively.

**Fig. 10.**
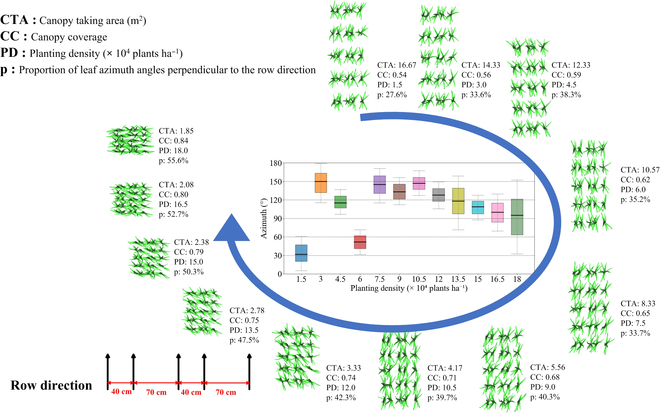
The visualization of the 12 planting density-gradient 3D maize canopy models and the characteristic distribution of leaf azimuth angles perpendicular to the row direction in each canopy. The center figure describes the proportion of leaf azimuth angles perpendicular to the row direction.

Using our method, we constructed twelve 3D maize canopy models and analyzed the distribution of azimuth angles of plants at each density (Fig. 10). The results revealed that up to a planting density of 9 × 10^4^ plants ha^−1^, the azimuth angle distribution of maize plants exhibited fluctuations and randomness. However, as the density surpassed this threshold, the overall azimuth angle gradually stabilized and tended toward 90°, i.e., perpendicular to the row direction. This trend is highly consistent with actual field observations of maize canopies. At lower planting densities, the competition for light resources among maize canopies is not intense, leading to a more random distribution of plants. Nevertheless, as plant density increases, to maximize the utilization of limited light resources, maize leaves adaptively adjust their distribution to be as perpendicular as possible to the row direction, thereby receiving more light. From a top-down perspective, one can visually observe how, at different planting densities, the distribution of maize canopies gradually becomes more ordered and aligned perpendicular to the row direction.

### Computational efficiency of the method

The entire computation process was conducted on a desktop computer equipped with an Intel i5-12700 CPU, 16GB RAM, and an NVIDIA GeForce RTX 3060 Ti GPU. Each iteration took approximately 12.33 s, and on average, it took about 7 h to complete the construction of a 3D model C_3_ for a canopy containing 25 plants. The original construction based on 3D phytomers takes up 2% of the time. Following this, the process moves to initially optimizing the 3D maize canopy model using CI, which accounts for a consequential 75% of the timeline. Subsequently, the focus shifts to involving the reflection-based optimization of the 3D maize canopy model, occupying 20% of the time. Finally, the project is dedicated to collision detection and response, comprising the remaining 3% of the overall duration.

**Fig. 11.**
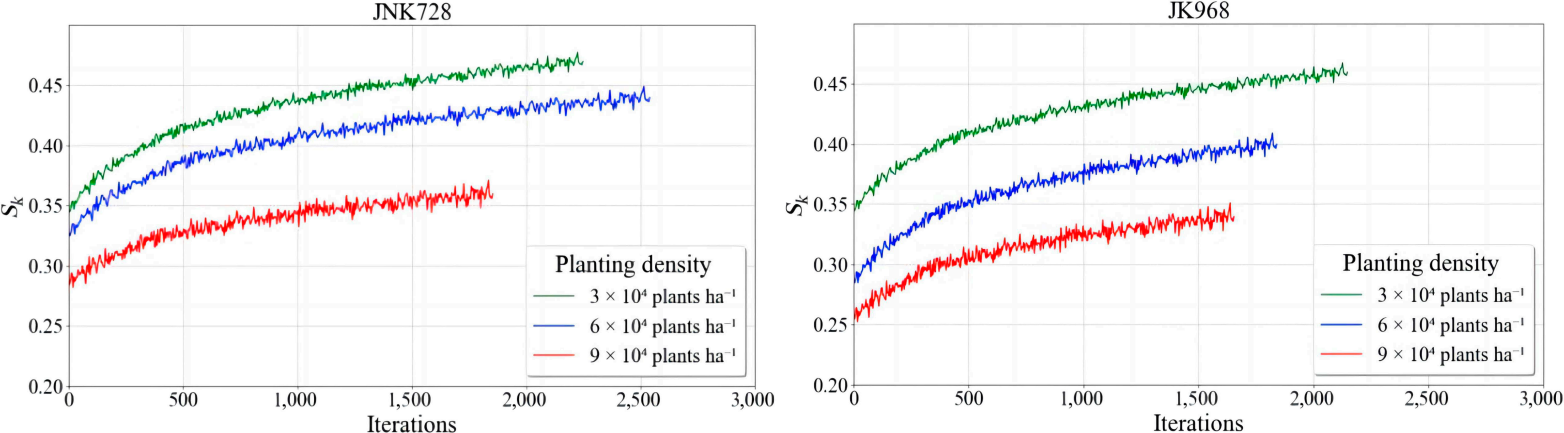
The dynamic curves of *S_k_* for different varieties and planting densities during the iterative process.

The core of 3D simulation of maize canopies based on CI focuses on the iterative process of optimizing and calculating the sunlit leaf area ratio index *S_k_* by adjusting the azimuth angles of the 3D phytomers. The objective is to progressively bring *S_k_* closer to its theoretical optimal value. To illustrate the computation process, Fig. 11 shows the trend of changes in *S_k_* with the number of iterations across 6 plots. The graph reveals that all 3D maize canopy models exhibit local fluctuations and an overall increasing trend during the optimization process, gradually stabilizing as the number of iterations increases. This aligns with the algorithm's design goal of finding the maximum convergent value of *S_k_*. The calculated value of *S_k_* for all canopy models fluctuates during the optimization process because changes in azimuth angle do not always guarantee an increase in *S_k_*. Thus, *S_k_* may experience minor fluctuations throughout the iterative process.

Additionally, there are distinct differences in the iterative process for the JNK728 and JK968 varieties at various planting densities. For instance, the JNK728 variety requires the fewest iterations at the highest planting density, while it needs the most iterations at medium planting density. Conversely, the JK968 variety requires the least number of iterations to converge at the highest planting density, but the most at the lowest planting density.

## Discussion

### Intelligent methods for efficient utilization of light resources in crop canopies

Canopies represent an organizational structure for life and production in extensive agricultural fields. The formation of these canopies is influenced by various factors such as planting density, varietal characteristics, environmental factors, and cultivation practices. Within these canopies, there is an abundance of morphological structures formed due to competition for resources among plants and their organs. This includes competition for water and nutrients by the root system and competition for light by the canopy leaves. Depicting this competition for resources within crop canopies is one of the challenges in agricultural science research.

Crop canopies can be considered as intelligent entities. However, research on swarm intelligence algorithms related to crops is scarce. For instance, inspired by the evolutionary behaviors of natural plant communities, researchers have developed a new type of wireless sensor network localization algorithm [38]. This algorithm mimics the processes of plant seeding, growth, and fruition, optimizing the accuracy of network node positioning through mathematical models, showing better adaptability and iterative effects than traditional algorithms. Zlobin et al. [39] proposed an algorithm to assess the vitality of individual plants, capable of analyzing and predicting the survival status of species within specific plant communities, providing crucial decision support for plant growth conditions. Additionally, Wang et al. [40] applied carnivorous plant predatory behaviors in algorithm design, introducing a method for solving the traveling salesman problem, which has shown consequential results in maintaining population diversity and improving solution quality.

However, there are currently few swarm intelligence algorithms specifically aimed at crops. Classic swarm intelligence algorithms, such as Particle Swarm Optimization, simulate the behavior of bird flocks to optimize spatial occupancy, while Ant Colony Optimization mimics ant foraging principles for path optimization. There is a lack of swarm intelligence algorithms focused on resource competition in crops. The primary idea of swarm intelligence is that each intelligent agent within the canopy achieves optimization and decision-making through mutual perception and computation. This study, focusing on the above-ground canopy structure of maize communities, proposes a 3D modeling algorithm for maize canopies. The core idea is that the 3D phytomers within the maize canopy are intelligent agents, and by intelligently adjusting their azimuth angles, the canopy captures more light, ultimately constructing 3D maize canopy models under different varieties, densities, and environmental conditions. This method can be considered a swarm intelligence algorithm aimed at efficient utilization of resources in 3D space, offering new perspectives for research on efficient resource utilization in crop canopies.

### Improvement for the construction of 3D maize canopy models

The 3D model of crop populations is a crucial component in the calculation of crop canopy light distribution based on 3D visualization, serving as an essential link between crop structure and function. The 3D model of crop populations greatly impacts subsequent calculations of canopy light distribution and photosynthetic productivity. Taking maize canopies as an example, the position of a leaf in the upper part of the canopy directly affects whether multiple organs in the lower part of the canopy can intercept direct sunlight. Currently, maize organs and plants can be accurately modeled in 3D through multiangle imaging or 3D scanning [25]. However, when generating 3D maize canopy models using actual measured plants, a random placement approach is still adopted [20,41]. To improve the consistency of 3D maize canopy models with actual field populations, the growth positions and orientations of plants within the population are determined through top-view images of the canopy, combined with the t-distribution method to integrate variety plant type information, thereby enhancing the consistency of 3D maize canopy models with actual field populations to some extent [9]. However, this method of constructing 3D maize canopy models struggles to present population characteristics caused by interorgan overlapping and resource competition within the population.

Based on our previous work, this paper proposes a 3D modeling of the maize canopy method aimed at efficient utilization of light resources. It optimizes the 3D maize canopy model by iteratively calculating each 3D phytomer within the population, combined with a reflective approach and collision detection and response. Given the high workload and low efficiency of high-precision acquisition of maize canopy 3D data [17], and the insufficient precision and automation level in maize canopy phenotypic analysis [15], this method enhances the mechanistic nature of 3D maize canopy model construction to some extent and can present the azimuth angle variation characteristics of maize canopies at different densities. Although there is still a difference between the constructed 3D maize canopy model and the actual field population, preventing a 1:1 3D reconstruction, it can still reflect population characteristics to a certain degree, thus promoting the construction of 3D maize canopy models and FSPMs research. For instance, this method constructs 3D models of maize populations with varying densities by obtaining the 3D plant architecture of maize varieties and combining the light environment of different eco-regions. The model considers the competition of organs within the population for light resources and the adjustment of positions caused by cross occlusion between organs, instead of using randomly copied individual plants. This method can be used for calculating canopy light distribution, radiation use efficiency, and crop production with population characteristics.

### Limitations and future work

This method relies on a maize 3D phytomer template library to reflect the variety characteristics of maize. Additionally, the computational efficiency of the method is relatively low. To narrow the iteration range, it is necessary to integrate more prior knowledge about maize morphological structure during the optimization process and incorporate relevant algorithms to enhance the intelligence level of the optimization process. The structure of crop populations is complex and influenced by many factors. This method has only considered light interception. Future work should integrate more maize canopy phenotypic information, maize growth knowledge models, and photosynthesis models. It is also essential to consider a wider range of environmental factors to improve the accuracy and practicality of the model.

## Data Availability

Data will be made available on request.
