# Supplementary material for: Three-Dimensional Modeling of Maize Canopies Based on Computational Intelligence
Source: Plant Phenomics. 2024 Mar 20;6:0160. doi: 10.34133/plantphenomics.0160 (PMC10950926; doi:10.34133/plantphenomics.0160)
Supplement: Supplementary 1 — Figs. S1 to S3 [file plantphenomics.0160.f1.zip › FigS2.pdf]

Algorithm Azimuth Iterative Optimization

$angle$ $Leaf\ Azimuth$
n $Maximum\ Iterations$
Qm $Maximum\ Convergence\ Difference$
I^{direct} $Intensity\ of\ Direct\ Solar\ Radiation$
lim $Convergence\ Threshold$

Input: Maize Population Data, Original Angle
Output: $max(S_{[i]}),\ angle(argmax(S_{[i]}))$

$i \leftarrow 0$
$N \leftarrow 0$
while $i < n$ or $N < lim$ **do**
  $angle \leftarrow renew(angle)$
  $S_{[i]} \leftarrow 0$
  for $j\ \ in\ \ enumerate(I^{direct})$ **do**
    $compute\ \ Direct\ Solar\ Radiation\ Leaf\ Area\ \ S_{[i,j]}$
    $S_{[i]} += S_{[i,j]}$
  end for
  $i++$
  if $i! = 0$ and $abs(S_{[i]} - S_{[i-1]}) < Qm$ **then**
    $N++$
  else
    $N = 0$
  end if
end while
