# Supplementary figures and images for: Three-Dimensional Modeling of Maize Canopies Based on Computational Intelligence
Source: Plant Phenomics. 2024 Mar 20;6:0160. doi: 10.34133/plantphenomics.0160 (PMC10950926; doi:10.34133/plantphenomics.0160)

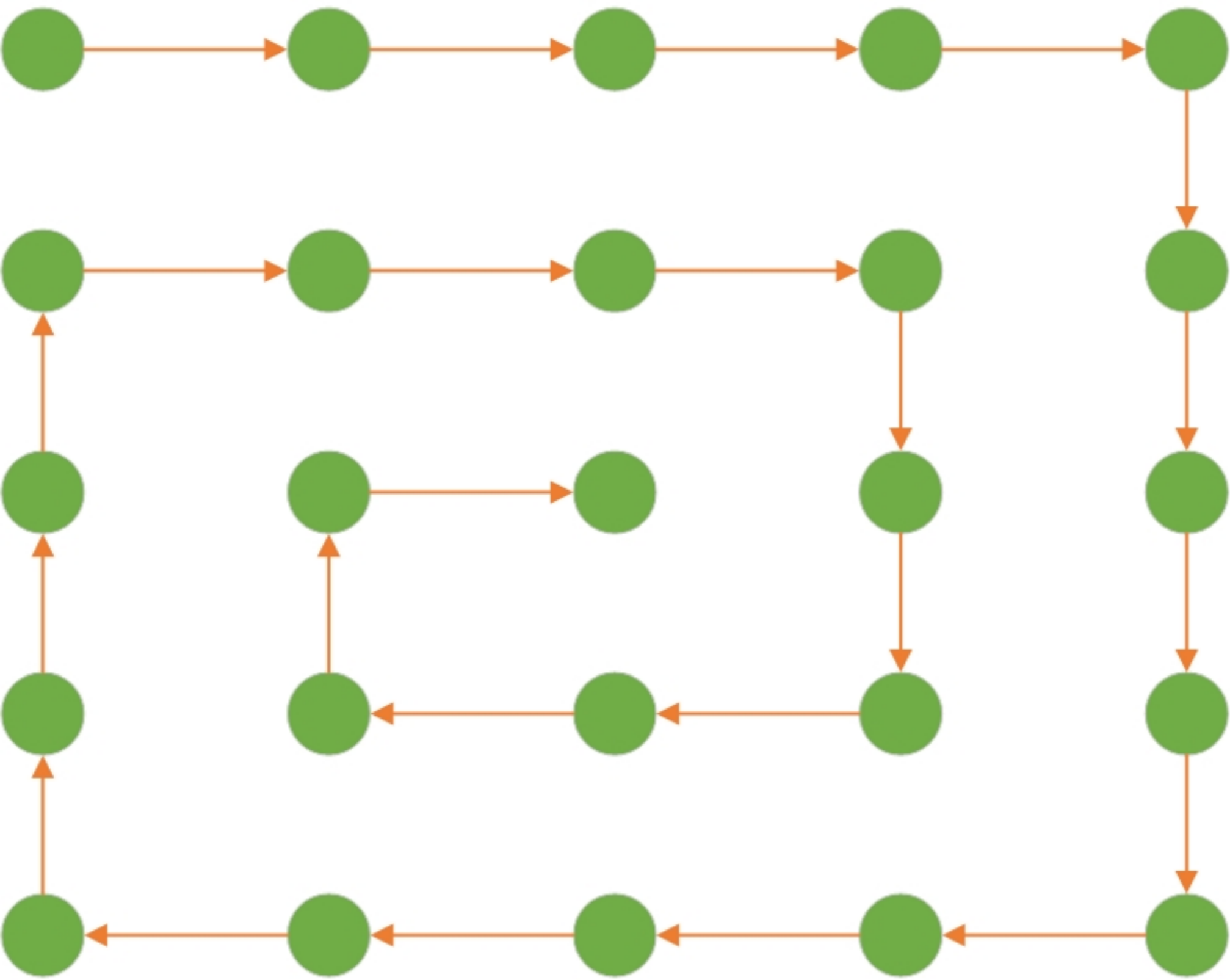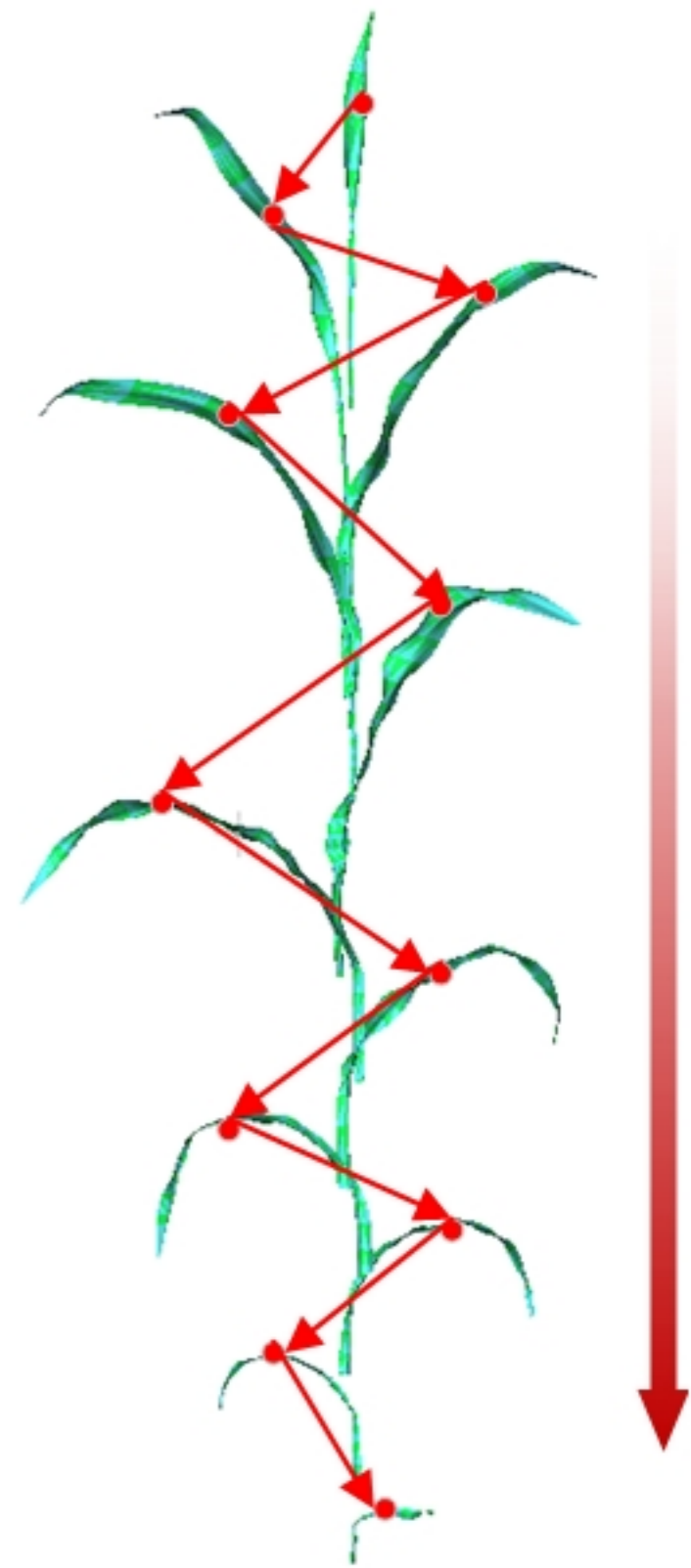

Supplement: Supplementary 1 — Figs. S1 to S3 [file plantphenomics.0160.f1.zip › FigS1.pdf]

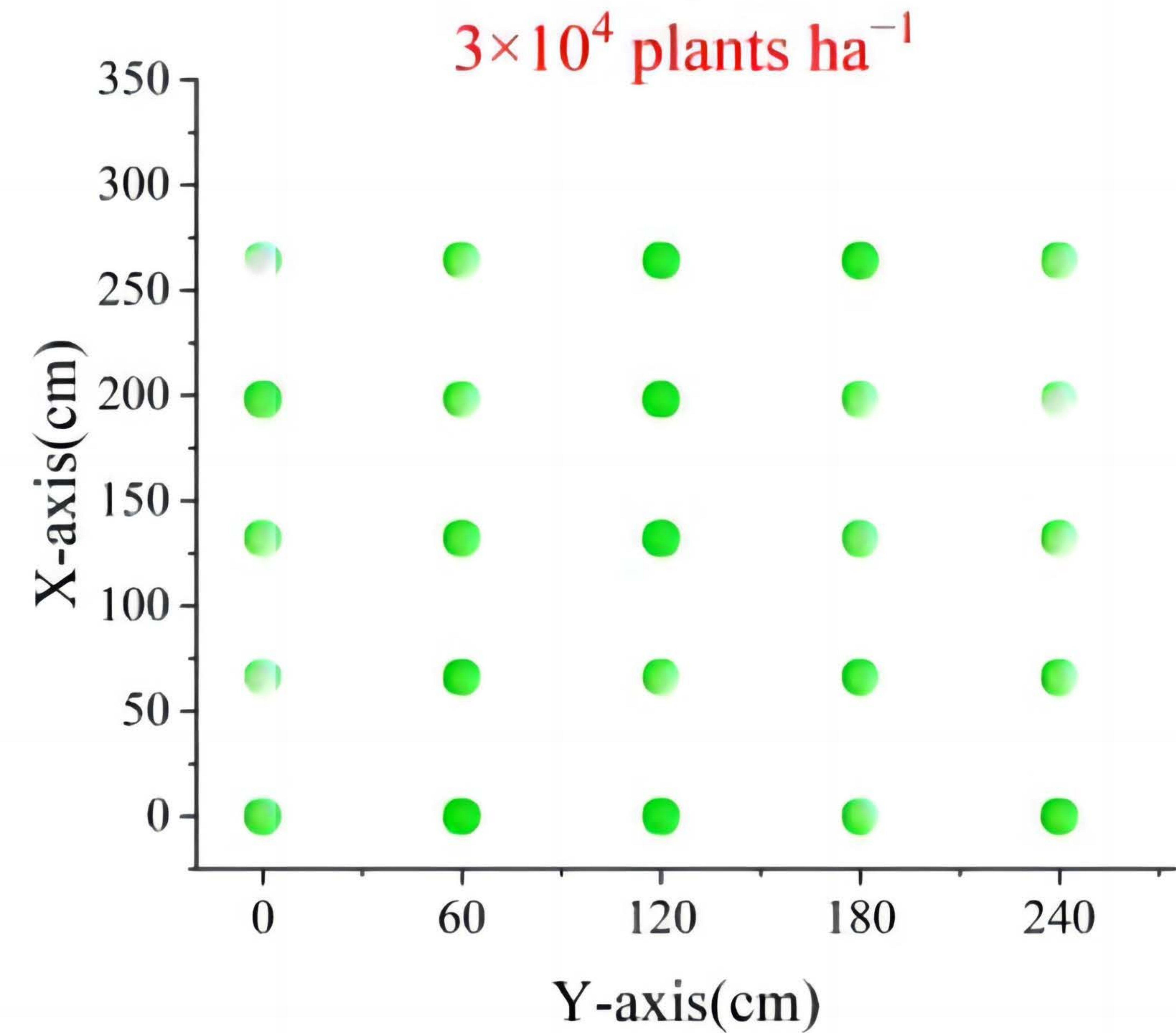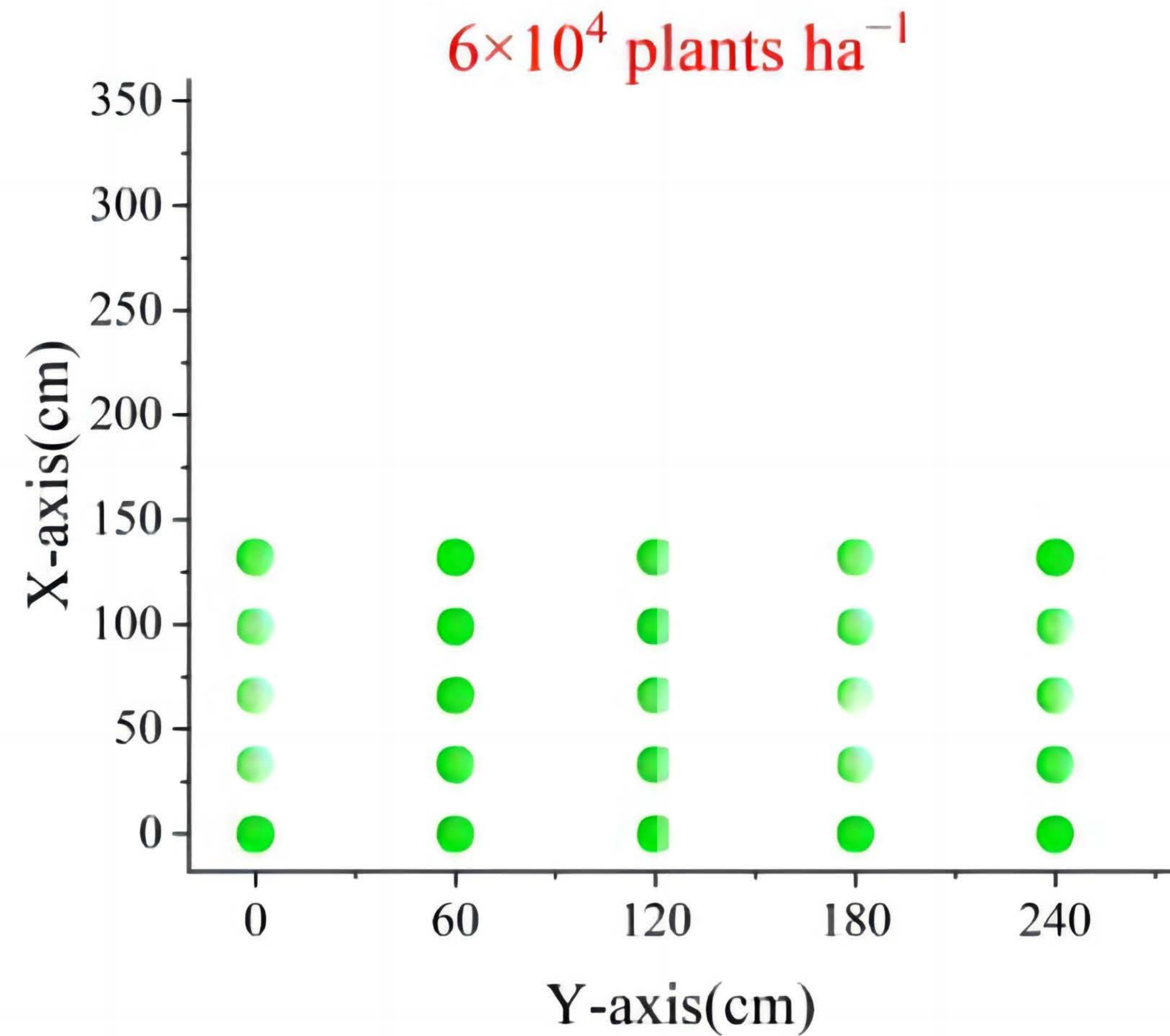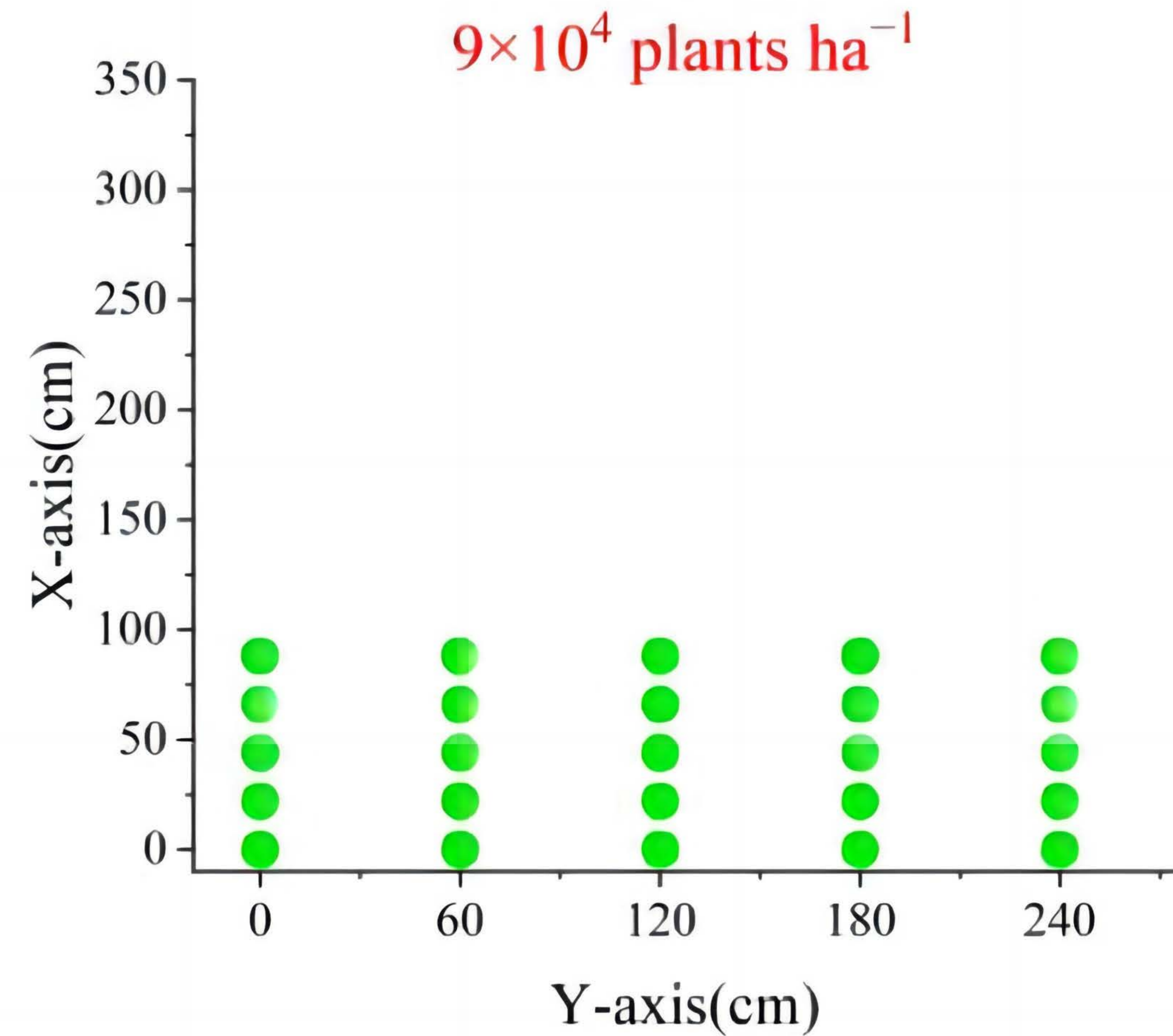

Supplement: Supplementary 1 — Figs. S1 to S3 [file plantphenomics.0160.f1.zip › FigS3.pdf]
