# Supplementary material for: Three-Dimensional Modeling of Maize Canopies Based on Computational Intelligence
Source: Plant Phenomics. 2024 Mar 20;6:0160. doi: 10.34133/plantphenomics.0160 (PMC10950926; doi:10.34133/plantphenomics.0160)
Supplement: Supplementary 1 — Figs. S1 to S3 [file plantphenomics.0160.f1.zip › Supplemental Material.doc]

(A)	(B)

**Fig. S1**. A schematic of the iterative adjustment order within the canopy (A), a schematic of the azimuth angle adjustment order within plants (B).

**Fig. S2**. The pseudo-code for the azimuth angle optimization iterative process.

**Fig. S3**. The schematic representation of maize plant positions in different density. The direction of increasing along the Y-axis is referred to as the row direction.
